# Exploring work ability, psychosocial job demands and resources of employees in low-skilled jobs: a German cross-sectional study

**DOI:** 10.1186/s12995-024-00429-2

**Published:** 2024-07-29

**Authors:** Arthur Kaboth, Lena Hünefeld, Marcel Lück

**Affiliations:** Federal Institute for Occupational Safety and Health, Friedrich-Henkel-Weg 1-25, 44149 Dortmund, Germany

**Keywords:** Low-skilled work, Job requirement level, COPSOQ, Working condition, Job-demand resources model, Interaction, Moderator

## Abstract

**Background:**

Extending working lives due to labour market and pension regulations makes maintaining and promoting work ability necessary. The coronavirus pandemic has shown that employees in low-skilled jobs (no qualification required) contribute significantly to society and the economy. Research on these employees has been neglected in Germany for many decades despite demanding working conditions. Therefore, we investigate the relationship between low-skilled jobs and work ability. Moreover, we explore this relationship’s variation by psychosocial work demands and resources.

**Methods:**

We use two waves of the German Study on Mental Health at Work (S-MGA). We calculate Ordinary-Least-Squares (OLS) regression models with pooled data (*n* = 6,050) to analyse the relationship between job requirement level and work ability. We also explore the contribution of job demands and resources on this relationship with interaction models. We use the Copenhagen Psychosocial Questionnaire (COPSOQ), to assess psychosocial work demands and resources.

**Results:**

Employees performing low-skilled jobs report significantly less work ability than those in medium- or high-skilled jobs. Interaction models show significantly greater work ability for employees in medium- and high-skilled jobs with high influence on their work (amount or tasks). Unexpectedly, employees in low-skilled jobs have lower work ability with more influence on their work. Furthermore, high role clarity, describing responsibility, authority and work goals, is associated with lower levels of work ability among employees in low-skilled jobs.

**Conclusions:**

The moderating effect of role clarity on the work ability of employees in low-skilled jobs can possibly be attributed to skills mismatch and limited responsibility, as well as a lack of self-perceived collective purpose of the job. The moderation of the influence on work dimension supports results of previous studies. Too much job autonomy can have negative effects under certain circumstances and is therefore perceived as a job demand in some studies. Consequently, mechanisms concerning psychosocial work demands and resources must be investigated in further studies with different theoretical approaches. The imbalance of job demands and resources shows that employers should invest in preserving the work ability to prevent early exit from the labour market in an aging society.

**Supplementary Information:**

The online version contains supplementary material available at 10.1186/s12995-024-00429-2.

## Background

The SARS-CoV-2 pandemic highlighted the importance of employees in low-skilled jobs for society and the economy. Low-skilled jobs describe activities for which no professional qualification is required [1, 2]. Despite the long research history, this group of employees has been neglected in the scientific community for decades. The current state of research on low-skilled work is still in need of further development but shows initial indications of an imbalance between work demands and resources: Employees in low-skilled work are often affected by high physical work demands and have few personal resources at their disposal [3, 4]. The work demands and resources of employees in medium- and high-skilled jobs, requiring completed vocational training or academic degrees, differ considerably from those of employees in low-skilled jobs. They have greater psychosocial work demands but more resources available [5]. Current results show an increase in psychosocial demands and work stress since the mid-1990s, especially among employees in low-skilled jobs [6].

To shed more light on psychosocial work demands and resources, and the supposed imbalance, we examine work ability by job requirement level, focusing on employees in low-skilled jobs. Work ability describes (individual) factors enabling workers to fulfil tasks successfully and emphasizes the discrepancy between demands and resources [7, 8]. It is considered a reliable predictor of other indicators, such as the timing of exiting the labor market or entering retirement, physical and mental health, sickness absence or disability [7, 9–16].

Studies show that resources help to promote and maintain work ability. According to Burr et al. [17], resources such as development opportunities or quality of leadership improve work ability. Airila et al. [18] demonstrated the long-term influence of personal and work-specific resources on work motivation and work ability. On the other hand, studies have shown that physical stress, e.g., the amount of work or the work pace, is associated with low work ability [17, 19].

However, literature reviews confirm that studies on work ability are predominantly concerned with employees in high-skilled jobs or specific sectors (e.g., the food industry or hospital employees) [17, 20]. Therefore, the present study investigates the work ability of employees in low-skilled jobs to address the imbalance of psychosocial work demands and resources.

A well-recognized theoretical model for analyzing this imbalance is the Job Demands-Resources model (JD-R). Work demands and resources can affect the individual or organizational level (e.g., personal health or company productivity) [21, 22]. According to Demerouti et al. [21], work demands include physical, social and organizational aspects (e.g., time/performance pressure). These factors are associated with psychological costs: The greater the work demands, the greater the associated costs. Long-lasting and extreme work demands lead to work stress. This describes the first of two processes of the JD-R model. Resources are understood as physical, psychological and social or organizational aspects of the activity (e.g., job security). They are needed to achieve specific (work) goals, promote personal development and learning processes, and reduce work demands [21, 22]. Permanently low resources lead to decreased motivation, describing the second process. Both processes are independent but influence each other so that specific resources can mitigate the negative consequences of particular work demands.

Firstly, we are interested in whether there are significant differences in work ability according to the job requirement level. Therefore, our first research question is: How do employees in low-, medium-, and high-skilled jobs differ in their levels of work ability? According to the JD-R model and the current state of research, employees in low-skilled jobs should have a significantly lower level of work ability than those in medium- and high-skilled jobs due to an imbalance in work demands and resources.

Secondly, we explore the role of psychosocial work demands and resources in the relationship between job requirement level and work ability by calculating interaction models. Our goal is to explore whether certain psychosocial job demands and resources moderate the effect on this relationship and pursue the following question: How do the differences in work ability vary by psychosocial work demands and resources? Our second research question visualizes the risks and opportunities for employees in low-, medium- and high-skilled jobs.

The analyses are carried out using two waves of the German Study on Mental Health at Work (S-MGA) [23, 24]. We analyze pooled data using linear regression models. For the second research question, we calculate interaction models to identify moderating effects of job demands and resources on the relationship between job requirement level and work ability.

Therefore, our study on work ability expands research on low-skilled work and can open up further research fields concerning this group of workers. Promoting and maintaining the work ability of employees in low-skilled jobs is a crucial objective, for example in combating early retirement. Furthermore, employees in these jobs are a resource to counteract the shortage of skilled labor in Germany, as three-quarters of employees in low-skilled jobs have completed vocational training.

## Methods

### Data source and sample

The German Study on Mental Health at Work (S-MGA) is a representative Panel study with two waves and includes employees aged 31 to 64, born between 1951 and 1980 (*N* = 7,148). The randomized sample of the study is derived from the Integrated Employment Biographies (IEB), a register data set of the German Federal Employment Agency. Information of participants on different topics were collected using Computer Assisted Personal Interviews (CAPI). The data set includes variables on working conditions, job demands, resources or different instruments, e.g. regarding depressive symptoms as the Patient Health Questionnaire (PHQ-9) [23, 24].

For the present study, we reduced the sample to employees with compulsory social security contributions who had complete information on the skill level of the job (*n* = 6,050). Therefore, civil servants, self-employed individuals and freelances are not included in the analysis. Due to the small number of employees in low-skilled work, the data from the 2011/2012 and 2017 waves must be pooled so that only cross-sectional analyses are possible.

### Measures

The variables used for the study are listed in Table A1 (see supplementary material). The table is intended to provide a better overview of all variables and to shed more light on the contents of the respective constructs. For this purpose, the individual questions of the respective constructs have been included in the table.

#### Dependent variable

Work ability, which describes (individual) factors enabling workers to fulfil tasks successfully [7, 8], is the dependent variable in the form of a single item. The self-assessment of those in employment ranges from “completely unable to work (0)” to “the best work ability ever achieved [10]”. Ebener & Hasselhorn [25] recommend using a single item on work ability in their validation study, as it correlates with the construct of work ability as an index and can be used for labor sciences.

#### Independent variable

In this study, low-, medium- and high-skilled jobs are defined from the job requirement level. Low-skilled jobs describes activities for which no professional training is required. Medium-skilled jobs, on the other hand, require professional training. For high-skilled jobs a master craftsman, technician training or an academic qualification is needed.

We operationalize these employment groups with the German Classification of Occupations from 2010 (KldB 2010). Unskilled/semiskilled tasks are understood as jobs in low-skilled work (*n* = 431). Skilled tasks are medium-skilled work (*n* = 3,190). High-skilled work (*n* = 2,429) comprise (highly) complex tasks.

#### Covariates

The covariates are socio-demographic characteristics such as age and whether an educational qualification is available. Gender was only included in the descriptive results, as it does not correlate with job requirement level or work ability. In addition, we control the survey wave to exclude periodic effects that might only be attributed to one of the two waves.

In this study, psychosocial work demands and resources are formed using the COPSOQ, an instrument for measuring psychosocial stress to initiate activities and to improve the psychosocial working environment [26]. This instrument is recognized in the scientific community, has been validated several times internationally and is consistent with the theoretical approaches of the JD-R model [27, 28]. The COPSOQ consists of several dimensions, each of which can be composed of several items [27]. The instrument is flexible in the selection of items, as the questionnaire can be adapted to the respective contexts (national, cultural or professional) [29]. We also add physical working conditions as a scale compromising 5 different items. Cronbach’s alpha is calculated for all scales and listed in the results section in Table 1.

For this study, the COPSOQ has eight dimensions, comprising various items from the survey. All items, for the COPSOQ and the physical working conditions, take on values between 1 (never / almost never, to a very low degree or strongly agree) and 5 (always, to a very high degree or disagree) and are totaled and averaged per person. High values mean a positive value for the individuals. For example, high values are synonymous with low demands or high resources. This applies to all scales of the items and dimensions.

### Statistical analyses

**Fig. 1:**
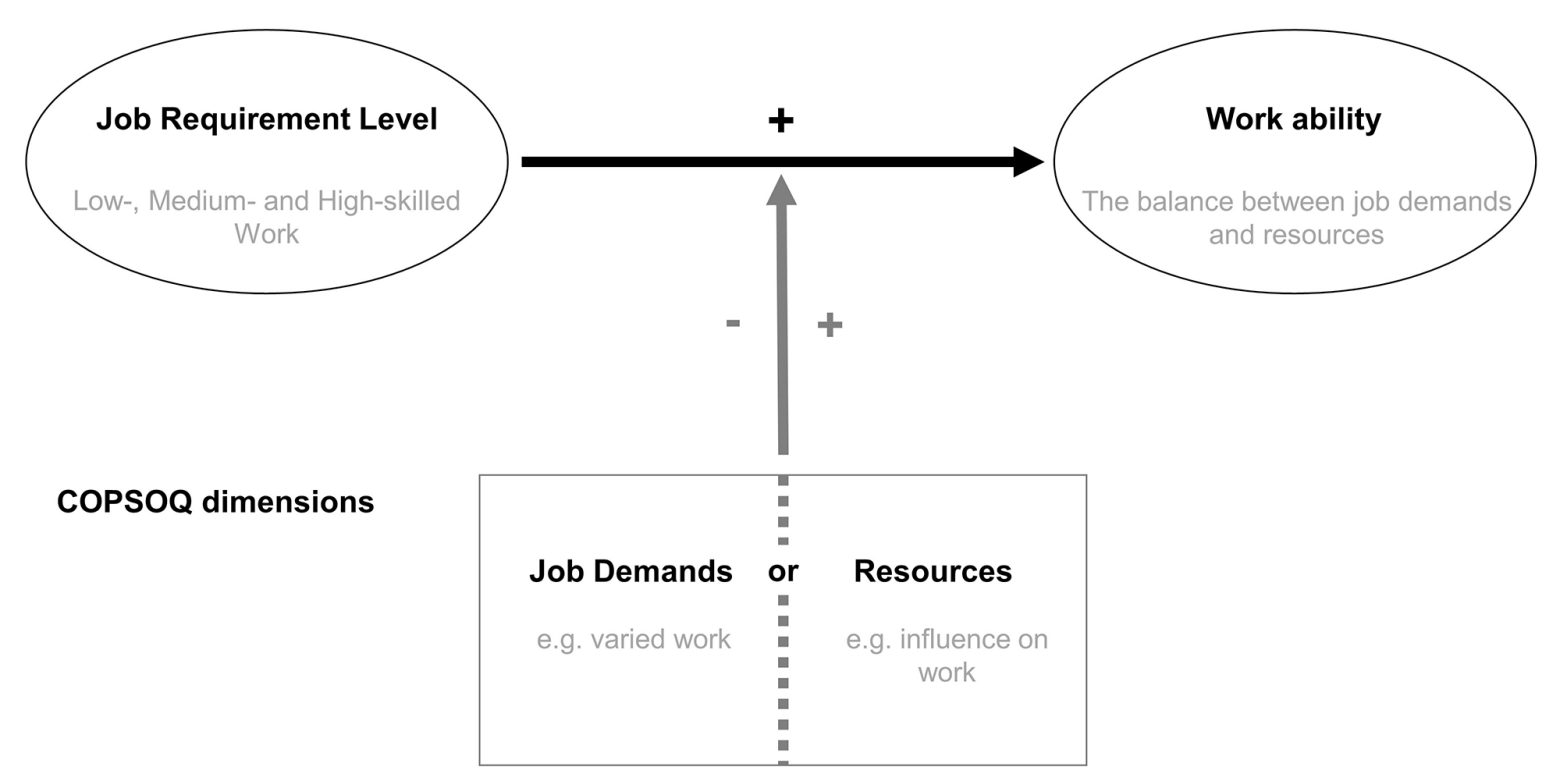
Moderation model of job demands or resources. Source: own presentation

Linear Ordinary-Least-Squares regressions (OLS) are calculated for the analyses [30, 31] to determine the relationship between job requirement level and work ability. Employees in low-skilled and medium-skilled are compared with those in high-skilled jobs. For all regression models, we conducted clustered robust standard errors to account for multiple observations from the same individual due to the pooled data set. The main models integrate various sociodemographic variables, work demands and resources according to the COPSOQ. We calculate interaction models (moderation) between the respective activity and the resources or work demands for our second research question. The model for the moderation can be seen in Fig. 1.

The metric variables used for the interaction effects are z-standardized (z) [32–34]. Post-hoc simple slope tests are conducted for significant interaction effects to (1) determine more precisely which values between independent and moderating variables interact with each other and (2) simultaneously facilitate the interpretation of the results [31, 32].

## Results

### Descriptive statistics

According to Table 1, most employees in low-skilled jobs are female (65.9%). This value is significantly higher than in the comparison groups. In addition, the proportion of employees with completed training or studies is lower in low-skilled work than in medium- and high-skilled work. However, a majority of 72.2% have completed training or have a degree.

**Table 1:** Descriptive statistics for employees in low-, medium-, and high-skilled jobs

	Low-skilled			Medium-skilled			High-skilled			Total			
	Mean/ %	SD		Mean/ %	SD		Mean/ %	SD		Mean/ %	SD	Cronbach alpha	
Age (Ø)	50.19	7.62		49.10	7.85		48.65	7.90		48.99	7.86		
Female (%)	65.90			52.00			42.70			49.30			
Training / studies completed (%)	72.20			96.00			99.20			95.60			
**Resources**													
Influence on work (Ø)	2.39	0.77		2.78	0.79		3.35	0.75		2.98	0.84	0.76	
Development possibilities (Ø)	2.90	0.92		3.64	0.77		4.08	0.59		3.76	0.78	0.69	
Job security (Ø)	3.23	1.13		3.53	1.04		3.78	0.96		3.61	1.03	0.49	
Role clarity (Ø)	4.22	0.61		4.27	0.57		4.28	0.58		4.27	0.58	0.67	
Social support (Ø)	3.23	0.82		3.44	0.75		3.41	0.70		3.42	0.74	0.79	
**Demands**													
Work-life conflict (Ø)	3.78	1.20		3.62	1.13		3.32	1.09		3.51	1.13	0.84	
Varied work (Ø)	3.08	1.43		4.00	1.11		4.28	0.82		4.05	1.08	-	
Quant. demands (Ø)	3.39	0.80		3.23	0.77		2.88	0.79		3.10	0.80	0.81	
Physical Working conditions (Ø)	3.29	0.66		3.55	0.54		3.83	0.41		3.64	0.53	0.73	
Work ability (Ø)	7.47	2.11		7.90	1.80		8.23	1.59		8.00	1.76		

Source: S-MGA 2011/2012 & 2017; own presentation and calculation, unweighted

Concerning the COPSOQ dimensions, the average value for the influence on work dimension, meaning for example the impact on the amount of work, is lower in low-skilled jobs than in the comparison groups. A similar tendency can be seen in the development opportunities dimension, where employees in low-skilled jobs have lower average values than the other groups. The average values for the job security dimension show that job security is lower among employees in low-skilled jobs than among those in the other groups. In the role clarity dimension, the differences between the groups are barely perceptible. In addition, social support from colleagues and the quality of the supervisor are rated worse by employees in low-skilled jobs than in the other two groups of employees.

With regard to work demands, the results of the work-life conflict dimension indicate fewer conflicts, on average, compared to the other two groups. Furthermore, employees in low-skilled jobs report, on average, less variety or, conversely, higher monotony. The dimension quantitative demands is greater in low-skilled jobs than for the other employee groups. This means that employees in low-skilled jobs have fewer quantitative demands. The descriptive statistics also show that employees in low-skilled jobs are often affected by physical working conditions.

Regarding work ability, employees in high-skilled jobs report the highest value. Medium-skilled work is just below the sample value. Employees in low-skilled jobs report the lowest work ability value. Overall, the descriptive results show that employees in low-skilled jobs have fewer resources available than those in other labor market groups. According to the JD-R, this could result in low work ability.

### Regression analyses

For the first research question of the study, we use the following regression model to find out whether there are differences between the occupational groups in terms of work ability, which describes the imbalance between job demands and resources. The reference group contains employees in high-skilled jobs. The results can be found in Table 2.

**Table 2:** Pooled OLS regression models for employees in low- and medium-skilled and work ability

	Work ability		
	Coef.	RSE	t
Low-skilled (Ref. High-skilled)	-0.311	0.125	-2.49*
Medium-skilled (Ref. High-skilled)	-0.202	0.052	-3.90***
Age	-0.031	0.003	-10.03***
Education	-0.052	0.136	-0.39
Survey wave	0.003	0.044	0.08
Quant. demands	0.134	0.035	3.76***
Influence on work	0.090	0.033	2.75**
Varied work	0.106	0.028	3.74***
Development possibilities	0.116	0.042	2.76**
Role clarity	0.221	0.047	4.72***
Job security	0.145	0.025	5.71***
Work-life conflict	0.159	0.025	6.25***
Social support	0.193	0.037	5.20***
Physical working conditions	0.200	0.052	3.88***
Intercept	4.714	0.350	13.48***
R2	0.12		
adj. R2	0.12		
N	5862		

Source: S-MGA 2011/2012 & 2017; own presentation and calculation, unweighted; *** *p* < .001, ** *p* < .01, * *p* < .05, Coef: Unstandardized Coefficient; RSE: Robust Standard Errors (clustered)

The regression model shows that employees in low-skilled jobs have significantly lower levels of work ability compared to those in high-skilled jobs (*r* = − .311, *p* < .05). Employees in medium-skilled jobs also have lower levels of work ability compared to those in high-skilled jobs (*r* = − .202, *p* < .001).

For the second research question, we explore the effects of job demands and resources on the relationship between job requirement level and work ability with interaction models. With the interaction models we want to explore whether certain psychosocial job demands and resources moderate the effect of the relationship. Table 3 shows the results with employees in high-skilled jobs as the reference group for the moderating effects of quantitative demands, influence on work, varied work and development possibilities.

**Table 3:** Interaction models of COPSOQ dimensions on the relationship of employees in low-skilled (LS) or medium-skilled jobs (MS) and work ability

	Quantitative Demands				Influence on Work				Varied Work				Development possibilities		
	Coef.	SE	t		Coef.	SE	t		Coef.	SE	t		Coef.	SE	t
Low-skilled (Ref. High-skilled)	-0.288	0.136	-2.11*		-0.386	0.135	-2.86**		-0.354	0.134	-2.64**		-0.342	0.147	-2.32*
Medium-skilled (Ref. High-skilled)	-0.199	0.052	-3.85***		-0.171	0.054	-3.18**		-0.187	0.053	-3.51***		-0.177	0.054	-3.28**
Age	-0.031	0.003	-10.03***		-0.031	0.003	-10.04***		-0.031	0.003	-10.04***		-0.031	0.003	-10.08***
Education	-0.053	0.136	-0.39		-0.044	0.136	-0.32		-0.039	0.136	-0.29		-0.047	0.136	-0.35
Survey wave	0.004	0.044	0.09		0.005	0.044	0.12		0.003	0.044	0.06		0.005	0.044	0.12
Quantitative Demands					0.137	0.035	3.86***		0.136	0.035	3.83***		0.135	0.035	3.79***
Quantitative Demands (z)	0.071	0.038	1.86												
Influence on Work	0.089	0.033	2.72**						0.089	0.033	2.72**		0.089	0.033	2.71**
Influence on Work (z)					0.161	0.042	3.87***								
Varied Work	0.106	0.028	3.74***		0.107	0.028	3.81***						0.107	0.028	3.79***
Varied Work (z)									0.187	0.051	3.64***				
Development possibilities	0.118	0.042	2.78**		0.122	0.042	2.89**		0.117	0.042	2.77**				
Development possibilities (z)													0.164	0.048	3.45***
Role Clarity	0.222	0.047	4.74***		0.215	0.047	4.62***		0.216	0.047	4.63***		0.217	0.047	4.65***
Job security	0.145	0.025	5.71***		0.145	0.025	5.72***		0.143	0.025	5.67***		0.144	0.025	5.71***
Work-life conflict	0.161	0.026	6.31***		0.159	0.025	6.27***		0.159	0.025	6.26***		0.161	0.025	6.31***
Social Relationships	0.192	0.037	5.17***		0.195	0.037	5.26***		0.193	0.037	5.20***		0.192	0.037	5.18***
Physical working conditions	0.197	0.052	3.78***		0.206	0.051	4.00***		0.201	0.051	3.91***		0.197	0.052	3.81***
LS # Quant. Quantitative Demands (z)	-0.012	0.131	-0.09												
MS # Quant. Quantitative Demands (z)	0.070	0.049	1.44												
LS # Influence on Work (z)					-0.261	0.129	-2.02*								
MS # Influence on Work (z)					-0.128	0.053	-2.44*								
LS # Varied Work (z)									-0.143	0.097	-1.48				
MS # Varied Work (z)									-0.088	0.060	-1.47				
LS # Development possibilities (z)													-0.129	0.107	-1.21
MS # Development possibilities (z)													-0.096	0.058	-1.66
Intercept	5.118	0.359	14.25***		4.890	0.356	13.75***		5.134	0.360	14.26***		5.149	0.360	14.32***
R2	0.12				0.12				0.12				0.12		
adj. R2	0.12				0.12				0.12				0.12		
N	5862				5862				5862				5862		
Source: S-MGA 2011/2012 & 2017; own presentation and calculation, unweighted; *** *p* < .001, ** *p* < .01, * *p* < .05															

**Fig. 2:**
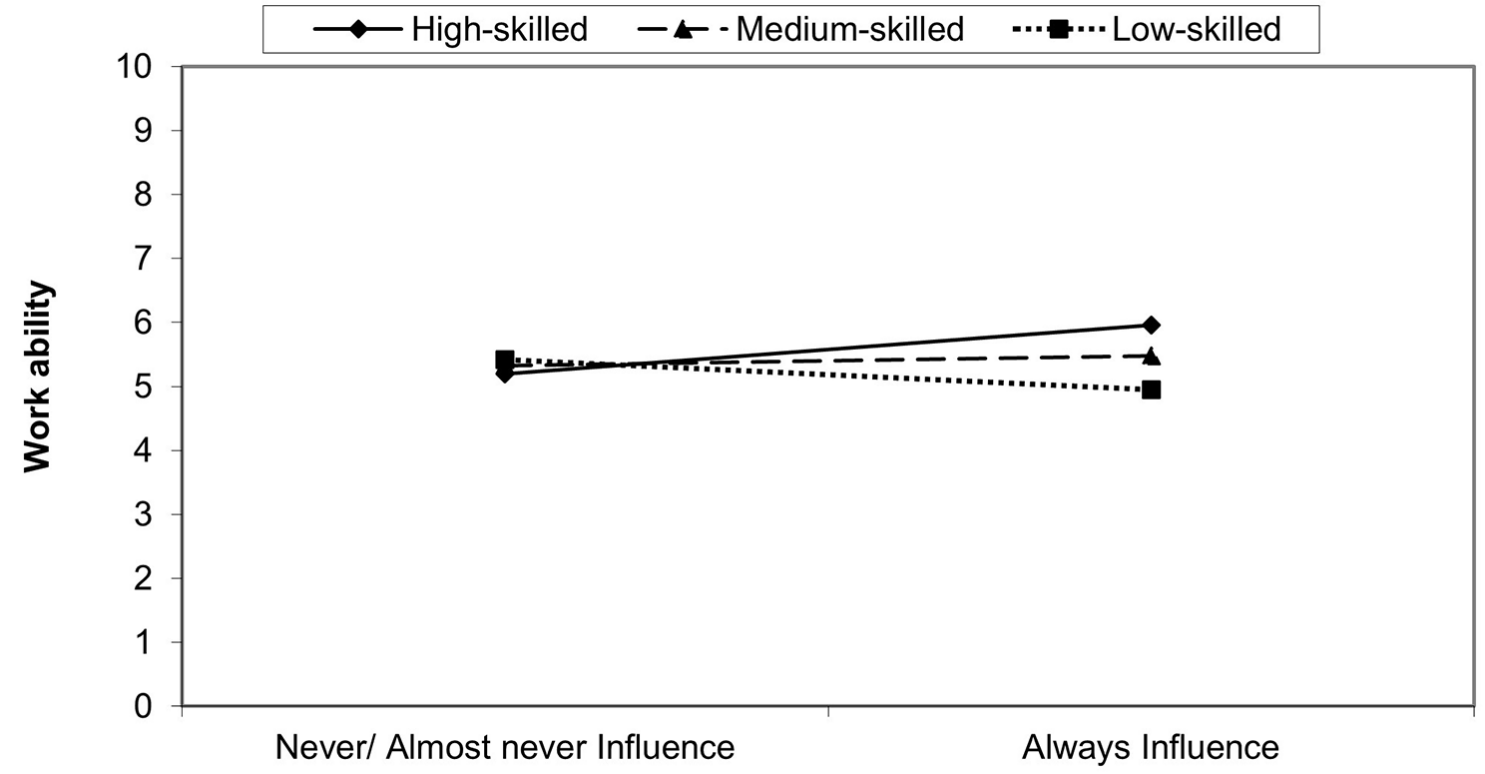
The interaction of the influence on work dimension. Source: S-MGA 2011/2012 & 2017; own presentation and calculation, unweighted

In Table 3, three out of four interactions on low-skilled or medium-skilled work and work ability are insignificant (quantitative demands, varied work and development possibilities). However, the interaction of the influence on work dimension, describing the influence for example on the amount or the tasks of work, is significant for employees in low-skilled and medium-skilled jobs. To simplify the interpretation of the effect of influence on work on the relationship between job requirement level and work ability, Fig. 2 illustrates the direction of the effects. Afterwards, a simple slope test is carried out.

Figure 2 shows that the level of influence on work is moderating the effect on work ability differently across job requirement level. Employees in low-skilled jobs have fewer work ability with more influence on their work compared to those who (almost) never have influence on their work. The opposite is true for employees performing medium- and high-skilled jobs: employees with high influence on their work report greater levels of work ability compared to those with less influence.

The simple slope test, in comparison of low- and high-skilled jobs, confirms a significant interaction among those with high levels of influence on their work (*r* = -1.013, *p* < .01). For employees with a low level of influence, the results are insignificant (*r* = .232; *p* > .05).

The simple slope test between medium- and high-skilled jobs confirms a significant moderation among those with high levels of influence on their work (*r* = − .480, *p* < .05). The test for those who never or almost never have influence on their work is insignificant (*r* = .133, *p* > .05).

Table 4 shows the moderating effects with the COPSOQ dimensions role clarity, job security, work-life conflict and social support.

**Table 4:** Interaction models of COPSOQ dimensions on the relationship of employees in low-skilled jobs (LS) or medium-skilled jobs (MS) and work ability

	Role Clarity			Job security			Work-life conflict			Social Support		
	Coef.	SE	t	Coef.	SE	t	Coef.	SE	t	Coef.	SE	t
Low-skilled (Ref. High-skilled)	-0.330	0.127	-2.61**	-0.338	0.129	-2.62**	-0.312	0.131	-2.38*	-0.316	0.122	-2.59**
Medium-skilled (Ref. High-skilled)	-0.202	0.052	-3.88***	-0.203	0.052	-3.88***	-0.202	0.052	-3.90***	-0.202	0.052	-3.87***
Age	-0.031	0.003	-10.12***	-0.031	0.003	-10.04***	-0.031	0.003	-10.04***	-0.031	0.003	-10.03***
Education	-0.047	0.136	-0.35	-0.049	0.135	-0.36	-0.052	0.136	-0.38	-0.052	0.136	-0.38
Survey wave	0.008	0.044	0.17	0.005	0.044	0.11	0.004	0.044	0.08	0.003	0.044	0.08
Quantitative Demands	0.131	0.035	3.71***	0.134	0.036	3.77***	0.134	0.036	3.76***	0.133	0.035	3.76***
Influence on Work	0.090	0.033	2.74**	0.091	0.033	2.76**	0.090	0.033	2.75**	0.090	0.033	2.75**
Varied Work	0.105	0.028	3.72***	0.105	0.028	3.74***	0.106	0.028	3.75***	0.106	0.028	3.75***
Development possibilities	0.116	0.042	2.76**	0.114	0.042	2.72**	0.116	0.042	2.75**	0.116	0.042	2.76**
Role Clarity				0.220	0.047	4.70***	0.221	0.047	4.71***	0.220	0.047	4.70***
Role Clarity (z)	0.171	0.039	4.44***									
Job security	0.142	0.025	5.61***				0.145	0.025	5.71***	0.144	0.025	5.71***
Job security (z)				0.134	0.039	3.47***						
Work-life conflict	0.161	0.025	6.31***	0.159	0.025	6.24***				0.159	0.025	6.25***
Work-life conflict (z)							0.175	0.041	4.33***			
Social Relationships	0.189	0.037	5.09***	0.193	0.037	5.19***	0.193	0.037	5.19***			
Social Relationships (z)										0.146	0.041	3.57***
Physical working conditions	0.201	0.051	3.90***	0.201	0.052	3.90***	0.199	0.052	3.84***	0.201	0.052	3.89***
LS # Role Clarity (z)	-0.221	0.109	-2.03*									
MS # Role Clarity (z)	-0.050	0.052	-0.96									
LS # Job security (z)				-0.047	0.102	-0.46						
MS # Job security (z)				0.035	0.050	0.69						
LS # Work-life conflict (z)							0.008	0.118	0.07			
MS # Work-life conflict (z)							0.008	0.050	0.16			
LS # Social Relationships (z)										-0.025	0.103	-0.24
MS # Social Relationships (z)										-0.003	0.052	-0.06
Intercept	5.688	0.324	17.54***	5.241	0.349	15.01***	5.273	0.362	14.55***	5.372	0.358	15.00***
R2	0.12			0.12			0.12			0.12		
adj. R2	0.12			0.12			0.12			0.12		
N	5862			5862			5862			5862		

Source: S-MGA 2011/2012 & 2017; own presentation and calculation, unweighted; *** *p* < .001, ** *p* < .01, * *p* < .05

**Fig. 3:**
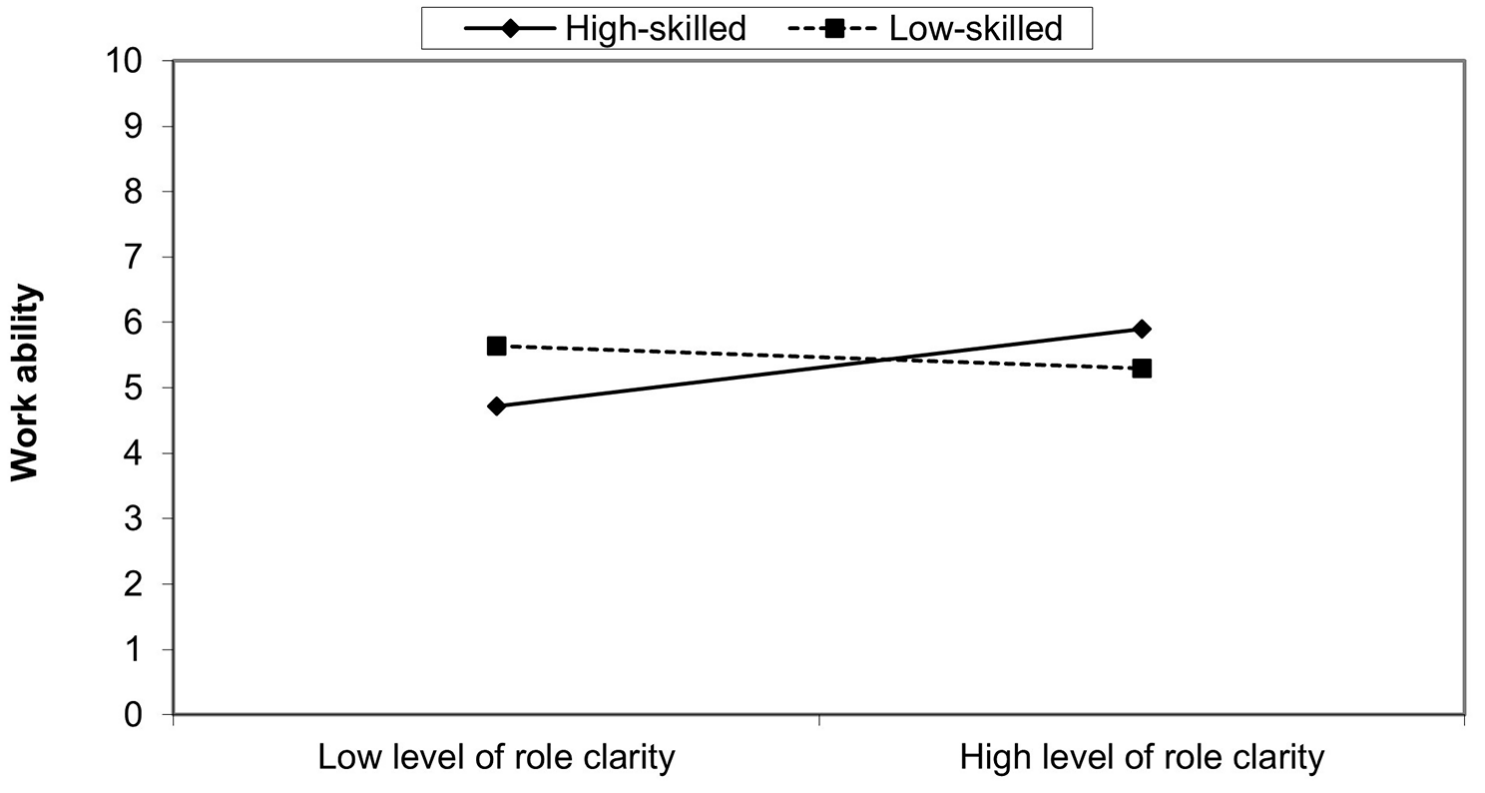
The interaction of the role clarity dimension. Source: S-MGA 2011/2012 & 2017; own presentation and calculation, unweighted

In Table 4, the interactions with job security, work-life conflict and social support are insignificant. Only the dimension of role clarity, describing the extent of clear goals, responsibility, and the knowledge of the extent of authority, shows a significant result regarding low-skilled work (*r* = − .221, *p* < .05). Figure 3 illustrates the direction of the effects and shows a differentiated relationship between role clarity, job requirement level and work ability. Employees in low-skilled jobs with low role clarity report greater work ability than those with high role clarity. The opposite is recognized for employees in high-skilled jobs: High levels of role clarity are associated with greater work ability.

The simple slope test only confirms a significant interaction between job requirement level and work ability among those who indicate a high degree of role clarity (*r* = − .610; *p* < .01). Among employees with a low level of role clarity, the results are not significant (*r* = .927; *p* > .05).

## Discussion

### Low-skilled work and work ability

The current state of research on employees in low-skilled jobs provides indications of an imbalance between work demands and resources [3]. Similarly, little is known about the work ability of employees in low-skilled jobs. Due to the suggested imbalance of demands and resources, this study investigates the relationship between job requirement level and work ability based on the JD-R model. Moreover, we are exploring whether certain psychosocial work demands and resources moderate this relationship.

Our first research question focuses on the relationship between job requirement level and work ability. Employees in low-skilled jobs report few quantitative demands and, at the same time, fewer resources, for example, in the dimensions of influence on work or social support. Ultimately, the OLS regression models confirmed the imbalance: low-skilled jobs are associated with low work ability. In contrast, employees in medium- and high-skilled jobs report greater levels of work ability.

### Influence on work as moderator on work ability

The second research question addresses the moderating effects of COPSOQ dimensions on the relationship between job requirement level and work ability. The COPSOQ dimension influence on work shows a positive moderation effect among employees in high- and medium-skilled jobs. This finding corresponds to the theoretical explanations according to the JD-R model [21, 22]. The greater the level of resources, the greater the work ability. Comparable results, even if occupational class is used as a moderator, can be found in the study by Lu et al. [35] on the relationship between autonomy and mental health. In particular, employees in high occupational positions benefit from workplace autonomy.

The dimension influence on work has a negative effect on the relationship between low-skilled jobs and work ability. Therefore, the result cannot fully be explained with the JD-R model. Our findings support the results of previous studies that influence on work or job autonomy can be seen as a stressor or demand under certain circumstances. Job autonomy requires planning and organization of work for which additional resources are needed [36]. In this context, Warr’s [37] vitamin model offers a coherent explanation for this result, as it assumes non-linear relationships. Many empirical results show non-linear effects especially concerning influence on work or job autonomy on different outcomes, for example job satisfaction (see 38, 39). According to Warr [40] job autonomy is detrimental in combination with certain job characteristics, such as time pressure or lower levels of social support. These are typical (job) characteristics of low-skilled work [3]. Further explanations are assumed by the “too-much-of-a-good-thing” effect [41] or the “choice overload” [42].

### The moderating effect of role clarity

The interaction with role clarity shows a significant moderating effect among employees performing low-skilled jobs. The reasons for the negative interaction effect among employees in low-skilled jobs have yet to be identified.

One possible explanation could be related to education or skill mismatch. Unlike in the 1990s, most people in low-skilled jobs have completed vocational training [43]. According to descriptive results, employees in low-skilled jobs are predominantly under-challenged by their work [1]. The knowledge of one’s limited area of responsibility and authority possibly leads to job dissatisfaction and lower levels of work ability.

A further explanation for the negative moderating effect of role clarity on work ability among employees in low-skilled jobs can be derived from the considerations of Jahoda’s [33] latent deprivation. In addition to a manifest function (income), work also fulfils latent functions. In this context, collective goals, i.e. the perception of the benefits of one’s activity for society and the job’s meaningfulness, could be central aspects. For employees in low-skilled jobs, greater role clarity could lead to realizing their low status and lack of meaning in their work. Sottimano et al. [44] reports that the meaning of work influences maintaining work ability especially among older employees. Furthermore, employees in lower-status occupations report significantly less collective purpose than those with higher occupational status [34]. Therefore, the perceived importance or meaningfulness of the job could explain the adverse interaction effect of role clarity.

Concerning the interaction with role clarity, the opposite applies to employees in high-skilled jobs: High role clarity is associated with high work ability. The result concerning high-skilled jobs is in line with previous studies on role clarity. According to Bliese & Castro [45] and Lang et al. [46], role clarity moderates the relationship between work demands and psychological and physical stress. A study by Orgambidez et al. [47] demonstrated the moderating effect of role clarity on the relationship between social support and job satisfaction. Firstly, the studies correspond to the theoretical considerations of the JD-R model. High resources mitigate the adverse effects of work demands. Secondly, the studies show that authority, responsibility and role assignments are essential in professions with human lives in focus (e.g. nurses and soldiers).

### Outlook and practical implications

Overall, the social perception of this group of employees increased significantly during the coronavirus pandemic but quickly declined. Research can make essential contributions to maintaining perception of this group. Therefore, further research is necessary and valuable. Against this background, the relationship between job requirement level and work ability in different occupational contexts should be investigated in the future, considering different working and organizational conditions. Greater focus should also be given to the individual characteristics of employees, for example: Might pre-existing health conditions make some people more susceptible to the adverse effects of low-skilled jobs or specific resources?

Against the background of current labor market challenges and retirement regulation, further research questions also open up in low-skilled jobs, as the work ability is considered a reliable predictor for retirement timing, labor market exit, and health or disability [7, 9–14]. The present study focused on the psychosocial work demands, which are distributed differently according to employee groups. However, more resources do not necessarily lead to a positive effect, as the results display. This and the insignificant interaction models should not lead to the conclusion that resources generally have no (positive) effect on the work ability of employees in low-skilled jobs. Results from Zolg and Herbig [36] for example show that the organizational and societal context is important for how the effect of resources such as autonomy unfolds. Therefore, further and specific research on those employees with different theoretical models is desirable. Concerning role clarity and influence on work, future studies should reflect the professional context. The combination of different resources and (physical) work demands should be analyzed. For example, the relationship between role clarity and job autonomy. The latter is classified as very low by employees in low-skilled jobs. This offers the possibility of using other in-depth theoretical approaches to consider work demands and resources in low-skilled jobs, such as the job-demand-control model [48], the conservation of resources theory [49], the vitamin model [37] or the latent deprivation model [50]. The processes of these models could be analyzed from a longitudinal perspective.

However, attention is also needed at the political and company levels. From occupational health and safety perspective, numerous tools are available that can help maintain or promote work ability or health and reduce stress. First and foremost, the work ability index or the risk assessment are tools for identifying problems and initiating solutions and should be emphasized. Employers should consider how to organize workplaces appropriately for employees in low-skilled work. These can include incorporating ergonomic principles, offering skill development opportunities, or increasing task variety. This could be accompanied by intervention studies to evaluate workplace design measures. Concerning role clarity and job autonomy as a moderator for the context analyzed here, it is difficult to derive suitable measures, as further and specific results are needed. At the very least, workplace health interventions that address organizational or time management issues could be considered to help reduce the stress of employees associated with job autonomy or role clarity.

### Limitations

The present study has limitations. The data could not be analyzed longitudinally due to the small number of cases of employees in low-skilled jobs. Accordingly, no causal or selective relationships could be analyzed. Similarly, the duration of the respective occupation or exposure to the work demands could not be considered. Furthermore, the S-MGA sample is limited to employees with social insurance contributions. Employees in marginal employment^1^ are underrepresented, but would have been of interest in the case of low-skilled jobs. In addition, the minimum age of the study sample is 31, meaning that employees under this age limit cannot be included. Low-skilled jobs can be found in all age groups [3].

## Conclusions

Employees in low-skilled jobs are associated with lower work ability than those in medium- or high-skilled jobs, reflecting the current discrepancy between job demands and resources. The interaction models show that the JD-R model is suitable to explain the variation in the relationship of job requirement level and work ability but not necessarily for employees in low-skilled jobs as there are adverse results. Further research should reflect different theoretical approaches and organizational as well societal factors to understand the mechanisms that involve these employees. In particular, employers should balance work demands and resources in a target group-oriented manner to ensure at least long-term preservation of work ability to cope with the extension of working lives, for example, using work ability index or risk assessments, especially for employees performing low-skilled jobs.

### Electronic supplementary material

Below is the link to the electronic supplementary material.

Supplementary Material 1

## Data Availability

The German Study on Mental Health at Work (S-MGA) is available upon application to the Federal Institute for Occupational Safety and Health at https://www.baua.de/EN/Research/Research-Data/Research-Data_node.html (accessed 28 May 2024).

## Footnotes

1 Marginal employment, also known as “mini-jobs”, refers to an employment relationship in which a low income can be earned without having to pay all social security contributions. At the time of the study presented here, this income limit was 400 euros.

## Abbreviations

S-MGA: Study on Mental Health at Work (Germany)
OLS: Ordinary–Least–Squares
COPSOQ: Copenhagen Psychosocial Questionnaire
JD-R: Job–Demands Resources
IEB: Integrated Employment Biographies
CAPI: Computer Assisted Personal Interview
PHQ-9: Patient Health Questionnaire
LS: Employees in low–skilled jobs
MS: Employees in medium–skilled jobs
