# Supplementary material for: Exploring work ability, psychosocial job demands and resources of employees in low-skilled jobs: a German cross-sectional study
Source: J Occup Med Toxicol. 2024 Jul 29;19:30. doi: 10.1186/s12995-024-00429-2 (PMC11285431; doi:10.1186/s12995-024-00429-2)
Supplement: Supplementary file 1 — Supplementary Material 1 [file 12995_2024_429_MOESM1_ESM.docx]

Table A1 Work ability, job requirement level, COPSOQ and physical demands dimensions, items and answer categories

| Dimension | Items |
| --- | --- |
| Work ability  (Dependent variable) | If you rate your best ever work ability at 10 points, how many points would you give your current work ability? Zero means that you are currently unable to work.  0 completely unable to work - 10 the best work ability ever achieved |
| Job requirement level  (Independent variable) | German Classification of Occupations from 2010 (5-digit)  Unskilled/ semiskilled tasks - Low-skilled work  Skilled tasks - Medium-skilled work  Complex tasks - High-skilled work  Highly complex tasks - High-skilled work |
| Quantitative demands  (COPSOQ) | "How often...   - do you have to work very fast?" - is your work unevenly distributed so that it piles up?" - does it happen that you don't have enough time to do all your tasks?" - do you fall behind on your work?" - do you have enough time for your work tasks?" - do you have to work overtime?"   Answer categories: 1 always - 5 (almost) never |
| Influence on work  (COPSOQ) | "How often...   - do you have a big impact on your work?" - do you influence who you work with?" - do you influence the amount of work that is assigned to you?" - do you influence what you do at work?" - can you decide for yourself when to take a break?" - are you more or less free to decide when to take a holiday?" - can you interrupt your work to talk to a colleague?" - can you leave your workplace for half an hour without special permission if you need to do private things?"   Answer categories: 1 (almost) never - 5 always |
| Varied work  (COPSOQ) | - "Is your work diversified?"   Answer categories: 1 (almost) never - 5 always |
| Development possibilities  (COPSOQ) | "To what extent...   - does your job require you to take the initiative?" - do you have the opportunity to learn new things through your work?" - can you apply your skills or expertise to your work?"   Answer categories: 1 to a very low degree - 5 to a very high degree |
| Role clarity  (COPSOQ) | "To what extent...   - do you know exactly how far your authority extends at work?" - are there clear goals for your work?" - do you know exactly what things are your responsibility?"   Answer categories: 1 to a very low degree - 5 to a very high degree |
| Job security  (COPSOQ) | "To what extent are you worried about/ do you worry that....   - becoming unemployed?" - it would be difficult for you to find a new job if you became unemployed?"   Answer categories: 1 to a very high degree - 5 to a very low degree |
| Work-life conflict  (COPSOQ) | - The time demands of my work make it difficult for me to fulfil my family or personal life responsibilities. - My work creates stress that makes it difficult to meet personal or family obligations. - Because of work commitments, I have to change plans for private or family activities.   Answer categories: 1 strongly agree - 5 disagree |
| Social support  (COPSOQ) | - How often do you receive help and support from your colleagues? - How often are your colleagues willing to listen to your work problems? - How often do your colleagues talk to you about the quality of your work? - To what extent does your immediate supervisor provide with good development opportunities for individual employees? - To what extent does your immediate supervisor place a high value on job satisfaction? - To what extent does your immediate supervisor plan work well? - To what extent does your immediate supervisor resolve conflicts well?   Answer categories: 1 (almost) never/ to a low degree - 5 always/ to a high degree |
| Physical working conditions | In each case, please assess how often these conditions apply to your activity.   - Working while standing - Working in a sitting position - Working bent over, squatting, kneeling, lying down or overhead - Lifting and/or carrying heavy loads (women more than 10 kg/ men more than 20 kg) - One-sided movements, in the sense of one-sided physical activity   Answer categories: 1 more than three quarters of the time, i.e., almost always – 5 never |

Source: S-MGA 2011/2012 & 2017; own presentation
